# Supplementary material for: Psychological Distress among University Staff before and during the COVID-19 Pandemic
Source: Int J Environ Res Public Health. 2023 Jan 26;20(3):2208. doi: 10.3390/ijerph20032208 (PMC9915217; doi:10.3390/ijerph20032208)
Supplement: Supplementary file 1 [file ijerph-20-02208-s001.zip › ijerph-2179558-supplementary.pdf]

Table S1. Stress Reaction among university staff before and during the COVID-19 pandemic.

	2019 Mean (SD)	2020 Mean (SD)	2021 Mean (SD)	*p*-Value [a]	Multiple Comparisons
**Time**	56.35 (14.62)	56.64 (14.59)	58.30 (15.58)	**< 0.001** ***	2019 vs. 2021 ***, 2020 vs. 2021 ***
**Time/sex**				**0.011** * [b]	
Time				**< 0.001** ***	2019 vs. 2021 ***, 2020 vs. 2021 ***
female	58.30 (14.54)	58.95 (14.55)	61.06 (15.62)	**< 0.001** ***	
male	54.30 (14.42)	54.22 (14.25)	55.41 (15.02)		
**Time/age**				**0.0011** ** [b]	
Time				**< 0.001** ***	2019 vs. 2021 ***, 2020 vs. 2021 ***
~29	58.97 (15.49)	60.16 (14.91)	63.55 (16.48)		
30–39	55.86 (14.02)	56.93 (14.15)	58.70 (15.34)		
40–49	57.30 (14.24)	57.06 (14.37)	58.83 (15.51)	**< 0.001** ***	
50–59	56.53 (15.00)	56.98 (15.12)	58.08 (15.06)		
60~	51.29 (14.11)	49.83 (12.43)	50.55 (13.88)		
**Time/occupation**				**< 0.001** *** [b]	
Time				**< 0.001** ***	2019 vs. 2020 *, 2019 vs. 2021 ***, 2020 vs. 2021 *
Office clerk	56.29 (14.68)	55.69 (14.15)	57.93 (15.41)		
Teacher	54.87 (14.76)	55.20 (15.26)	55.80 (15.43)		
Nurse	60.53 (14.11)	62.22 (13.60)	65.15 (15.52)		
Allied health Professional [c]	56.75 (14.38)	59.51 (15.21)	60.86 (15.16)	**< 0.001** ***	
Doctor	54.31 (10.22)	55.73 (12.14)	54.02 (10.73)		
Other	52.17 (15.11)	53.41 (13.30)	54.85 (14.33)		

[a] *p*-value was calculated using repeated measures of one or two-way ANOVA. If the sphericity test was significant, the *p*-value was adjusted using the Greenhouse-Geisser. Multiple comparisons were performed using Bonferroni's method. [b] This indicated the interaction of *p*-value. [c] Occupational therapists, physical therapists, speech therapists, orthoptists, pharmacists, clinical psychologists, radiological technologists, medical technologists, clinical engineering technologists, registered dietitians, and dental hygienists. * *p*-value < 0.05, ** *p*-value < 0.01, *** *p*-value < 0.001. *P*-values denoted in bold are significant at <0.05. ANOVA, analysis of variance; SD, standard deviation.

Table S2. Job Stressor among university staff before and during the COVID-19 pandemic.

	2019 Mean (SD)	2020 Mean (SD)	2021 Mean (SD)	*p*-Value [a]	Multiple Comparisons
**Time**	40.40 (6.85)	40.38 (6.98)	40.84 (7.12)	**< 0.001** ***	2019 vs. 2021 **, 2020 vs. 2021 ***
**Time/ sex**				**< 0.001** *** [b]	
Time				**< 0.001** ***	2019 vs. 2021 **, 2020 vs. 2021 ***
female	40.49 (6.89)	41.00 (7.02)	41.53 (6.87)	**0.002** **	
male	40.30 (6.81)	39.73 (6.87)	40.12 (7.31)		
**Time/ age**				**< 0.001** *** [b]	
Time				**< 0.001** ***	2019 vs. 2021 **, 2020 vs. 2021 ***
~29	42.18 (7.00)	43.45 (7.03)	44.75 (6.53)		
30–39	39.83 (7.06)	40.45 (6.73)	41.09 (7.17)		
40–49	40.67 (6.55)	40.26 (6.73)	40.74 (6.81)	**< 0.001** ***	
50–59	40.37 (6.84)	40.27 (7.23)	40.24 (7.18)		
60~	39.06 (6.69)	37.52 (6.27)	37.82 (6.60)		
**Time/ occupation**				**< 0.001** *** [b]	
Time				**0.011** *	2019 vs. 2021 *
Office clerk	38.88 (6.72)	38.53 (6.65)	39.16 (6.78)		
Teacher	40.01 (6.62)	39.81 (6.94)	39.89 (7.01)		
Nurse	44.39 (5.86)	45.48 (5.63)	46.32 (5.63)		
Allied health Professional [a]	43.53 (6.13)	43.83 (5.98)	44.40 (6.41)	**< 0.001** ***	
Doctor	42.54 (6.07)	42.50 (4.78)	42.31 (6.38)		
Other	40.27 (7.89)	41.17 (6.74)	41.56 (6.12)		

[a] *p*-value was calculated using repeated measures of one or two-way ANOVA. If the sphericity test was significant, the *p*-value was adjusted using the Greenhouse-Geisser. Multiple comparisons were performed using Bonferroni's method. [b] This indicated the interaction of *p*-value. [c] Occupational therapists, physical therapists, speech therapists, orthoptists, pharmacists, clinical psychologists, radiological technologists, medical technologists, clinical engineering technologists, registered dietitians, and dental hygienists. * *p*-value < 0.05, ** *p*-value < 0.01, *** *p*-value < 0.001. *P*-values denoted in bold are significant at <0.05. ANOVA, analysis of variance; SD, standard deviation.

Table S3. Social Support among university staff before and during the COVID-19 pandemic.

	2019 Mean (SD)	2020 Mean (SD)	2021 Mean (SD)	*p*-Value [a]	Multiple Comparisons
**Time**	19.61 (5.31)	19.99 (5.34)	20.50 (5.33)	**< 0.001** ***	2019 vs. 2020 ***, 2019 vs. 2021 ***, 2020 vs. 2021 ***
**Time/sex**				0.271 [b]	
Time				**< 0.001** ***	2019 vs. 2020 ***, 2019 vs. 2021 ***, 2020 vs. 2021 ***
female	19.34 (5.19)	19.86 (5.18)	20.35 (5.23)	0.114	
male	19.90 (5.41)	20.13 (5.51)	20.66 (5.42)		
**Time/age**				0.457 [b]	
Time				**< 0.001** ***	2019 vs. 2020 **, 2019 vs. 2021 ***, 2020 vs. 2021 ***
~29	17.54 (4.99)	18.27 (4.79)	18.91 (4.84)	**< 0.001** ***	
30–39	18.83 (5.11)	19.30 (5.34)	19.91 (5.31)		
40–49	19.95 (5.22)	20.36 (5.11)	20.76 (5.38)		
50–59	20.48 (5.39)	20.81 (5.61)	21.19 (5.40)		
60~	20.49 (5.35)	20.31 (5.31)	21.06 (5.09)		
**Time/occupation**				**0.013** * [b]	
Time				**< 0.001*****	2019 vs. 2020 **, 2019 vs. 2021 ***
Office clerk	19.84 (5.15)	20.06 (5.33)	20.49 (5.15)	0.271	
Teacher	19.66 (5.64)	19.95 (5.67)	20.61 (5.72)		
Nurse	18.54 (5.09)	19.51 (4.78)	20.31 (5.09)		
Allied health Professional [a]	20.36 (4.81)	20.88 (5.23)	20.71 (5.39)		
Doctor	19.15 (5.03)	19.52 (4.89)	20.21 (4.88)		
Other	20.59 (5.68)	21.29 (5.34)	20.44 (5.53)		

[a] *p*-value was calculated using repeated measures of one or two-way ANOVA. If the sphericity test was significant, the *p*-value was adjusted using the Greenhouse-Geisser. Multiple comparisons were performed using Bonferroni's method. [b] This indicated the interaction of *p*-value. [c] Occupational therapists, physical therapists, speech therapists, orthoptists, pharmacists, clinical psychologists, radiological technologists, medical technologists, clinical engineering technologists, registered dietitians, and dental hygienists. * *p*-value < 0.05, ** *p*-value < 0.01, *** *p*-value < 0.001. *P*-values denoted in bold are significant at <0.05. ANOVA, analysis of variance; SD, standard deviation.

Table S4. Multiple regression analyses of university staff before and during the COVID-19 pandemic.

	**Sex**	**Age**	**Teacher**	**Nurse**	**Allied Health Professional** [a]	**Doctor**
**Stress Reaction**	**0.10434033 *****	**−0.00207996 ****	0.00382886	**0.10061478 *****	**0.02906036 ***	−0.00329807
p-value	< 0.001	0.002	0.814	< 0.001	0.046	0.821
**Job Stressor**	−0.01830489	**−0.00141556 ***	**0.06978821 *****	**0.32815990 *****	**0.14648395 *****	**0.08559520 *****
p-value	0.210	0.030	< 0.001	< 0.001	< 0.001	< 0.001
**Social Support**	−0.00983467	**0.00723365 *****	**−0.04342033 ****	0.01150463	**0.03704861 ***	−0.02311090
p-value	0.520	< 0.001	0.008	0.480	0.011	0.115

[a] Occupational therapists, physical therapists, speech therapists, orthoptists, pharmacists, clinical psychologists, radiological technologists, medical technologists, clinical engineering technologists, registered dietitians, and dental hygienists. We demonstrate the standard partial regression coefficient. Dummy variables: occupation, office clerk = 0 and all other occupations = 1; sex, male = 0 and female= 1; year, 2019 = 0 and all other years = 1. * *p*-value < 0.05, ** *p*-value < 0.01, *** *p*-value < 0.001. The standard partial regression coefficient denoted in bold are significant at <0.05.

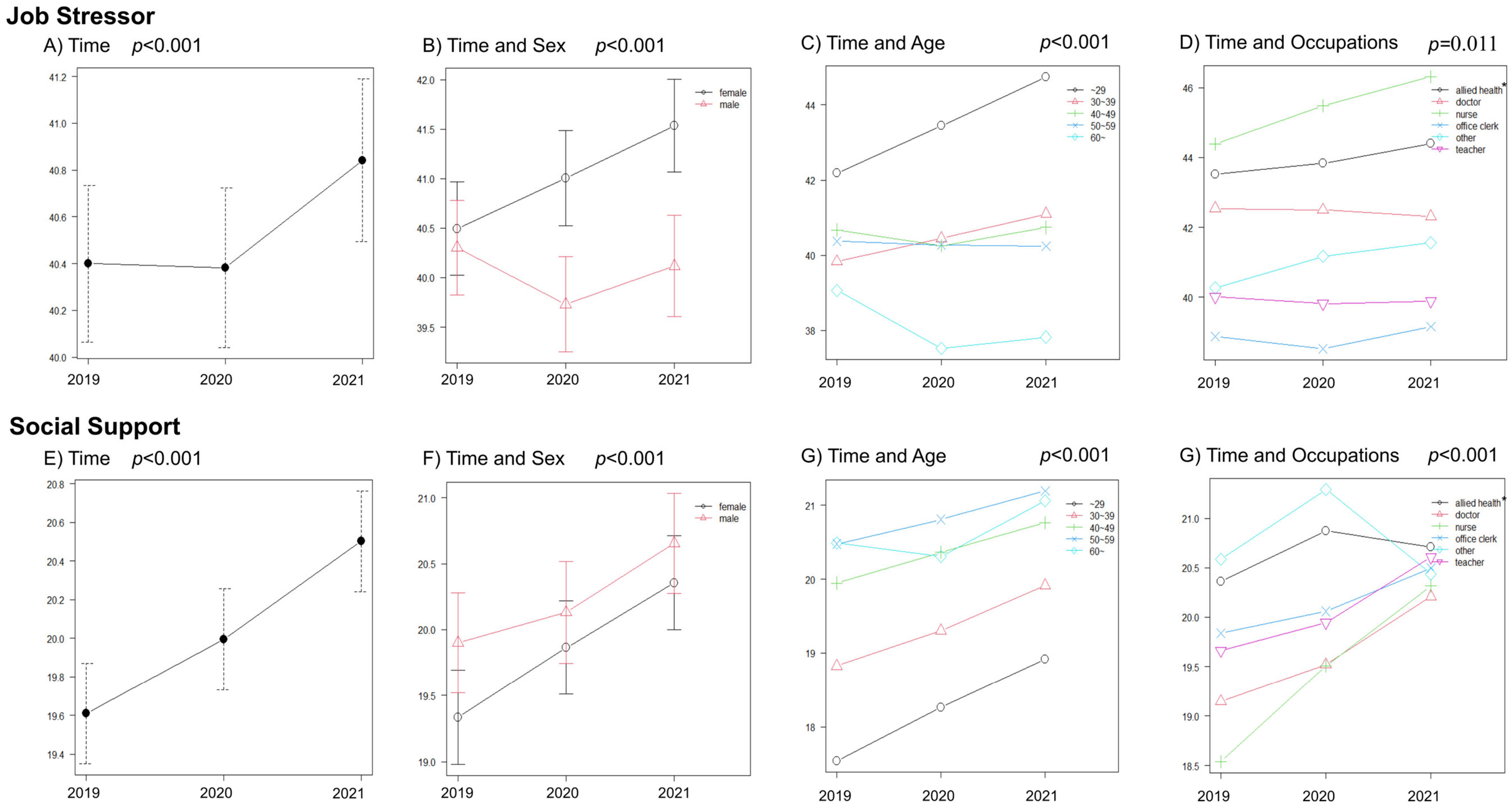

Figure S1. Job Stressor and Social Support among university staff before and during the COVID-19 pandemic.Job Stressor and Social Support are components of the BJSQ. Job Stressor and Social Support demonstrate (A)~(D) and (E)~(H), respectively. (A)/(E) Time, (B)/(F) Time and Sex, (C)/(G) Time and age, and (D)/(H) Time and Occupations. Line graphs demonstrate changes in Job Stressor and Social Support over time, and *p*-values of Time's main effects were obtained using repeated measures of two-way ANOVA. Error bars indicate 95% CI. * allied health = allied health professional ANOVA, analysis of variance; BJSQ, brief job stress questionnaire; CI, confidence interval; COVID-19, coronavirus disease-2019.

The Brief Job Stress Questionnaire English version

Please answer the following questions concerning your job by circling the number that best fits your situation.

	Very much so	Moderately so	Somewhat	Not at all
1. I have an extremely large amount of work to do	1	2	3	4
2. I can't complete work in the required time	1	2	3	4
3. I have to work as hard as I can	1	2	3	4
4. I have to pay very careful attention	1	2	3	4
5. My job is difficult in that it requires a high level of knowledge and technical skill	1	2	3	4
6. I need to be constantly thinking about work throughout the working day	1	2	3	4
7. My job requires a lot of physical work	1	2	3	4
8. I can work at my own pace	1	2	3	4
9. I can choose how and in what order to do my work	1	2	3	4
10. I can reflect my opinions on workplace policy	1	2	3	4
11. My knowledge and skills are rarely used at work	1	2	3	4
12. There are differences of opinion within my department	1	2	3	4
13. My department does not get along well with other departments	1	2	3	4
14. The atmosphere in my workplace is friendly	1	2	3	4
15. My working environment is poor (e.g. noise, lighting, temperature, ventilation)	1	2	3	4
16. This job suits me well	1	2	3	4
17. My job is worth doing	1	2	3	4

Please answer the following questions concerning your health during the past month by circling the number that best fits your situation.

	Almost never	Sometimes	Often	Almost always
18. I have been very active	1	2	3	4
19. I have been full of energy	1	2	3	4
20. I have been lively	1	2	3	4
21. I have felt angry	1	2	3	4
22. I have been inwardly annoyed or aggravated	1	2	3	4
23. I have felt irritable	1	2	3	4
24. I have felt extremely tired	1	2	3	4
25. I have felt exhausted	1	2	3	4
26. I have felt weary or listless	1	2	3	4
27. I have felt tense	1	2	3	4
28. I have felt worried or insecure	1	2	3	4
29. I have felt restless	1	2	3	4
30. I have been depressed	1	2	3	4
31. I have thought that doing anything was a hassle	1	2	3	4
32. I have been unable to concentrate	1	2	3	4

33. I have felt gloomy	1	2	3	4
34. I have been unable to handle work	1	2	3	4
35. I have felt sad	1	2	3	4
36. I have felt dizzy	1	2	3	4
37. I have experienced joint pains	1	2	3	4
38. I have experienced headaches	1	2	3	4
39. I have had a stiff neck and / or shoulders	1	2	3	4
40. I have had lower back pain	1	2	3	4
41. I have had eyestrain	1	2	3	4
42. I have experienced heart palpitations or shortness of breath	1	2	3	4
43. I have experienced stomach and / or intestine problems	1	2	3	4
44. I have lost my appetite	1	2	3	4
45. I have experienced diarrhea and / or constipation	1	2	3	4
46. I haven't been able to sleep well	1	2	3	4

Please answer the following questions concerning people around you by circling the number that best fits your situation.

	Extremely	Very much	Somewhat	Not at all
How freely can you talk with the following people?				
47. Superiors	1	2	3	4
48. Co-workers	1	2	3	4
49. Spouse, family, friends, etc.	1	2	3	4
How reliable are the following people when you are troubled?				
50. Superiors	1	2	3	4
51. Co-workers	1	2	3	4
52. Spouse, family, friends, etc.	1	2	3	4
How well will the following people listen to you when you ask for advice on personal matters?				
53. Superiors	1	2	3	4
54. Co-workers	1	2	3	4
55. Spouse, family, friends, etc.	1	2	3	4

Please answer the following questions concerning satisfaction by circling the number that best fits your situation.

	Satisfied	Somewhat satisfied	Somewhat dissatis-fied	Dissatisfied
56. I am satisfied with my job	1	2	3	4
57. I am satisfied with my family life	1	2	3	4
